# Supplementary material for: Validating metabarcoding-based biodiversity assessments with multi-species occupancy models: A case study using coastal marine eDNA
Source: PLoS One. 2020 Mar 19;15(3):e0224119. doi: 10.1371/journal.pone.0224119 (PMC7082047; doi:10.1371/journal.pone.0224119)
Supplement: S1 Table — List of studies (n = 5) that have examined occupancy models in the context of DNA metabarcoding. To obtain this list of publications, we performed the following systematic, Boolean search using the Web of Science: “*DNA” AND "metabarcoding" AND "occupancy model*". (DOCX) [file pone.0224119.s001.docx]

**S1 Table. Literature review of occupancy modeling for metabarcoding data.** List of studies (*n* = 5) that have examined occupancy models in the context of DNA metabarcoding. To obtain this list of publications, we performed the following systematic, Boolean search using the Web of Science: “*DNA” AND "metabarcoding" AND "occupancy model*".

| **Citation** | **System** | **Taxonomic group** | **Multi-Species?** | **Multi-Scale?** | **Gene** | **Primers** | **Read length (bp)** | **Mean # Reads / replicate** |
| --- | --- | --- | --- | --- | --- | --- | --- | --- |
| Ficetola et al. 2015 [1] | Simulations / Soil / Lake sediment | Lumbricidae / Bovidae | N | Y | 16S, mDNA | ewE / ewD | ~70 | Not reported |
| Ficetola et al. 2016 [2] | Perspective paper | Perspective paper | N/A | N/A | N/A | N/A | N/A | N/A |
| Lopes et al. 2016 [3] | Tropical streams | Anura (40 species total, 3 focal species) | N/A | N/A | 12S, rRNA | batra_F / batra_R | 50-54 | 9,186 |
| Valentini et al. 2016 [4] | Freshwater ponds and lakes | Amphibia / Osteichthyes | Y | N | 12S, mDNA | batra_F / batra_R | >20 | 253,626 |
| Doi et al. 2019 [5] | Aquarium tank | Osteichthyes / Chondrichthyes (168 species) | Y | Y | 12S, rRNA | MiFish-U / E | 163-185 | Not reported |
| This study | Coastal Marine | Metazoa | Y | Y |  |  |  |  |

References

1. Ficetola GF, Pansu J, Bonin A, Coissac E, Giguet-Covex C, De Barba M, et al. Replication levels, false presences and the estimation of the presence/absence from eDNA metabarcoding data. Mol Ecol Resour. 2015;15: 543–556. doi:10.1111/1755-0998.12338

2. Ficetola GF, Taberlet P, Coissac E. How to limit false positives in environmental DNA and metabarcoding? Mol Ecol Resour. 2016;16: 604–607. doi:10.1111/1755-0998.12508

3. Lopes CM, Sasso T, Valentini A, Dejean T, Martins M, Zamudio KR, et al. eDNA metabarcoding: a promising method for anuran surveys in highly diverse tropical forests. Mol Ecol Resour. 2017;17: 904–914. doi:10.1111/1755-0998.12643

4. Valentini A, Taberlet P, Miaud C, Civade R, Herder J, Thomsen PF, et al. Next-generation monitoring of aquatic biodiversity using environmental DNA metabarcoding. Mol Ecol. 2016;25: 929–942. doi:10.1111/mec.13428

5. Doi H, Fukaya K, Oka S, Sato K, Kondoh M, Miya M. Evaluation of detection probabilities at the water-filtering and initial PCR steps in environmental DNA metabarcoding using a multispecies site occupancy model. Sci Rep. 2019;9: 1–8. doi:10.1038/s41598-019-40233-1
