# Supplementary material for: Validating metabarcoding-based biodiversity assessments with multi-species occupancy models: A case study using coastal marine eDNA
Source: PLoS One. 2020 Mar 19;15(3):e0224119. doi: 10.1371/journal.pone.0224119 (PMC7082047; doi:10.1371/journal.pone.0224119)
Supplement: S2 Table — Species-specific estimates of occupancy, capture and detection using mean covariates values (water depth (m) and sequencing depth) from a multi-scale, multi-species occupancy model for eDNA metabarcoding data from Conception Bay, Newfoundland. (DOCX) [file pone.0224119.s002.docx]

**S2 Table.** **Species-specific parameter estimates from multi-species, multi-scale occupancy model.** Species-specific estimates of occupancy, capture and detection using mean covariates values (water depth (m) and sequencing depth) from a multi-scale, multi-species occupancy model for eDNA metabarcoding data from Conception Bay, Newfoundland.

| Species | Phylum | Occupancy | Probability of Capture | Probability of Detection |
| --- | --- | --- | --- | --- |
| Acrochaetium alariae |  | 0.20 | 0.95 | 0.04 |
| Acrochaetium moniliforme |  | 0.23 | 0.96 | 0.35 |
| Acrochaetium parvulum |  | 0.14 | 0.98 | 0.76 |
| Acrochaetium secundatum |  | 0.14 | 0.98 | 0.38 |
| Aeolidia papillosa | Mollusca | 0.32 | 0.95 | 0.05 |
| Agarum clathratum | Phaeophyceae | 0.63 | 0.97 | 0.08 |
| Aglantha digitale | Cnidaria | 0.30 | 0.95 | 0.05 |
| Ahnfeltia borealis |  | 0.22 | 0.96 | 0.03 |
| Ahnfeltia plicata |  | 0.14 | 0.98 | 0.58 |
| Alaria esculenta | Phaeophyceae | 0.09 | 0.97 | 0.22 |
| Alces americanus | Chordata | 0.22 | 0.95 | 0.04 |
| Alitta virens | Annelida | 0.15 | 0.81 | 0.73 |
| Amathia gracilis | Bryozoa | 0.18 | 0.95 | 0.15 |
| Ammodytes dubius | Chordata | 0.75 | 0.96 | 0.07 |
| Ammodytes hexapterus | Chordata | 0.09 | 0.97 | 0.31 |
| Amphibalanus improvisus | Arthropoda | 0.92 | 0.98 | 0.13 |
| Amphitrite figulus | Annelida | 0.34 | 0.86 | 0.41 |
| Ampithoe rubricata | Arthropoda | 0.08 | 0.97 | 0.34 |
| Ancula gibbosa | Mollusca | 0.20 | 0.95 | 0.04 |
| Anomalocera patersoni | Arthropoda | 0.20 | 0.95 | 0.04 |
| Arenicola marina | Annelida | 0.23 | 0.96 | 0.37 |
| Ascophyllum nodosum | Phaeophyceae | 0.31 | 0.90 | 0.57 |
| Asterias rubens | Echinodermata | 0.64 | 0.98 | 0.15 |
| Audouinella hermannii |  | 0.23 | 0.95 | 0.03 |
| Aurelia aurita | Cnidaria | 0.92 | 0.97 | 0.18 |
| Bonnemaisonia hamifera |  | 0.26 | 0.98 | 0.16 |
| Boreophyllum birdiae |  | 0.20 | 0.95 | 0.05 |
| Bos taurus | Chordata | 0.20 | 0.95 | 0.05 |
| Calliopius laeviusculus | Arthropoda | 0.14 | 0.98 | 0.65 |
| Callithamnion corymbosum |  | 0.85 | 0.98 | 0.23 |
| Cancer irroratus | Arthropoda | 0.14 | 0.98 | 0.94 |
| Caprella mutica | Arthropoda | 0.15 | 0.96 | 0.24 |
| Caprella septentrionalis | Arthropoda | 0.31 | 0.95 | 0.05 |
| Ceramium virgatum |  | 0.28 | 0.86 | 0.75 |
| Cheilotrichia cinerascens | Arthropoda | 0.15 | 0.96 | 0.08 |
| Chlorarachnion reptans |  | 0.20 | 0.95 | 0.11 |
| Chorda filum | Phaeophyceae | 0.87 | 0.63 | 0.53 |
| Chordaria chordaeformis | Phaeophyceae | 0.08 | 0.97 | 0.54 |
| Chordaria flagelliformis | Phaeophyceae | 0.92 | 0.98 | 0.34 |
| Chrysymenia wrightii |  | 0.20 | 0.97 | 0.10 |
| Cirratulus cirratus | Annelida | 0.74 | 0.96 | 0.04 |
| Clava multicornis | Cnidaria | 0.20 | 0.97 | 0.11 |
| Clione limacina | Mollusca | 0.65 | 0.94 | 0.10 |
| Clitellio arenarius | Annelida | 0.12 | 0.97 | 0.13 |
| Clupea harengus | Chordata | 0.73 | 0.90 | 0.14 |
| Clupea pallasii | Chordata | 0.19 | 0.95 | 0.05 |
| Cochliopodium kieliense |  | 0.21 | 0.95 | 0.05 |
| Coelocladia arctica | Phaeophyceae | 0.23 | 0.95 | 0.33 |
| Colaconema daviesii |  | 0.15 | 0.98 | 0.24 |
| Convoluta convoluta | Xenacoelomorpha | 0.28 | 0.97 | 0.14 |
| Corallina officinalis |  | 0.32 | 0.94 | 0.47 |
| Craterolophus convolvulus | Cnidaria | 0.15 | 0.93 | 0.10 |
| Ctenosciara hyalipennis | Arthropoda | 0.11 | 0.97 | 0.14 |
| Cyanea capillata | Cnidaria | 0.86 | 0.95 | 0.19 |
| Cyclosa conica | Arthropoda | 0.20 | 0.95 | 0.04 |
| Cylindrotheca closterium | Bacillariophyta | 0.24 | 0.96 | 0.07 |
| Cyrtodaria siliqua | Mollusca | 0.60 | 0.96 | 0.06 |
| Cystoclonium purpureum |  | 0.17 | 0.92 | 0.18 |
| Dendrodrilus rubidus | Annelida | 0.28 | 0.95 | 0.05 |
| Dendronotus frondosus | Mollusca | 0.20 | 0.95 | 0.04 |
| Devaleraea ramentacea |  | 0.17 | 0.93 | 0.16 |
| Dexamine thea | Arthropoda | 0.23 | 0.92 | 0.58 |
| Dictyosiphon ekmanii | Phaeophyceae | 0.24 | 0.87 | 0.39 |
| Dictyosiphon foeniculaceus | Phaeophyceae | 0.36 | 0.95 | 0.40 |
| Dodecaceria concharum | Annelida | 0.58 | 0.88 | 0.38 |
| Doto coronata | Mollusca | 0.30 | 0.96 | 0.09 |
| Drepanosiphum oregonense | Arthropoda | 0.33 | 0.97 | 0.07 |
| Dumontia contorta |  | 0.15 | 0.96 | 0.06 |
| Echinarachnius parma | Echinodermata | 0.32 | 0.97 | 0.09 |
| Ectocarpus crouaniorum | Phaeophyceae | 0.21 | 0.95 | 0.05 |
| Ectocarpus fasciculatus | Phaeophyceae | 0.08 | 0.97 | 0.41 |
| Ectocarpus siliculosus | Phaeophyceae | 0.15 | 0.94 | 0.25 |
| Ectopleura larynx | Cnidaria | 0.11 | 0.95 | 0.12 |
| Enchelyopus cimbrius | Chordata | 0.21 | 0.95 | 0.04 |
| Enchytraeus albidus | Annelida | 0.22 | 0.95 | 0.04 |
| Ephemerella subvaria | Arthropoda | 0.20 | 0.95 | 0.05 |
| Escharella immersa | Bryozoa | 0.32 | 0.95 | 0.04 |
| Eualus pusiolus | Arthropoda | 0.63 | 0.96 | 0.05 |
| Euthora cristata |  | 0.20 | 0.95 | 0.04 |
| Evadne nordmanni | Arthropoda | 0.41 | 0.97 | 0.09 |
| Florenciella parvula |  | 0.92 | 0.99 | 0.60 |
| Fucus distichus | Phaeophyceae | 0.15 | 0.98 | 0.20 |
| Fucus vesiculosus | Phaeophyceae | 0.74 | 0.96 | 0.31 |
| Fujientomon dicestum | Arthropoda | 0.73 | 0.96 | 0.06 |
| Gadus morhua | Chordata | 0.20 | 0.97 | 0.10 |
| Gammarus duebeni | Arthropoda | 0.20 | 0.95 | 0.05 |
| Gammarus lawrencianus | Arthropoda | 0.08 | 0.97 | 0.33 |
| Gammarus oceanicus | Arthropoda | 0.14 | 0.98 | 0.42 |
| Gammarus setosus | Arthropoda | 0.21 | 0.90 | 0.13 |
| Gasterosteus aculeatus | Chordata | 0.21 | 0.95 | 0.11 |
| Gasterosteus wheatlandi | Chordata | 0.15 | 0.96 | 0.06 |
| Glycera dibranchiata | Annelida | 0.14 | 0.95 | 0.32 |
| Gnathostomula armata | Gnathostomulida | 0.21 | 0.94 | 0.04 |
| Goniada maculata | Annelida | 0.76 | 0.97 | 0.20 |
| Grammonema striatula | Bacillariophyta | 0.36 | 0.90 | 0.36 |
| Halichondria panicea | Porifera | 0.18 | 0.98 | 0.11 |
| Halisarca dujardini | Porifera | 0.17 | 0.97 | 0.16 |
| Halocladius variabilis | Arthropoda | 0.14 | 0.92 | 0.58 |
| Halosiphon tomentosus | Phaeophyceae | 0.16 | 0.98 | 0.17 |
| Haslea ostrearia | Bacillariophyta | 0.75 | 0.97 | 0.22 |
| Haslea pseudostrearia | Bacillariophyta | 0.40 | 0.97 | 0.09 |
| Hecatonema maculans | Phaeophyceae | 0.14 | 0.98 | 0.50 |
| Hildenbrandia rubra |  | 0.14 | 0.98 | 0.53 |
| Homo sapiens | Chordata | 0.92 | 0.98 | 0.28 |
| Hydatophylax argus | Arthropoda | 0.20 | 0.95 | 0.05 |
| Hydrobates leucorhous | Chordata | 0.24 | 0.95 | 0.03 |
| Idotea baltica | Arthropoda | 0.28 | 0.96 | 0.06 |
| Ischyrocerus anguipes | Arthropoda | 0.30 | 0.95 | 0.05 |
| Jassa marmorata | Arthropoda | 0.45 | 0.91 | 0.40 |
| Lacuna vincta | Mollusca | 0.45 | 0.79 | 0.52 |
| Laminaria digitata | Phaeophyceae | 0.42 | 0.96 | 0.06 |
| Laminariocolax aecidioides | Phaeophyceae | 0.17 | 0.97 | 0.14 |
| Laodicea undulata | Cnidaria | 0.11 | 0.97 | 0.10 |
| Laomedea flexuosa | Cnidaria | 0.16 | 0.97 | 0.17 |
| Laonice cirrata | Annelida | 0.38 | 0.97 | 0.18 |
| Leathesia difformis | Phaeophyceae | 0.19 | 0.96 | 0.11 |
| Lepidonotus squamatus CMC02 | Annelida | 0.21 | 0.97 | 0.09 |
| Leucoraja ocellata | Chordata | 0.30 | 0.95 | 0.05 |
| Limacina helicina | Mollusca | 0.86 | 0.96 | 0.08 |
| Lithothamnion glaciale |  | 0.84 | 0.96 | 0.32 |
| Littorina littorea | Mollusca | 0.14 | 0.86 | 0.49 |
| Littorina obtusata | Mollusca | 0.15 | 0.90 | 0.37 |
| Littorina saxatilis | Mollusca | 0.08 | 0.97 | 0.42 |
| Lizzia blondina | Cnidaria | 0.47 | 0.95 | 0.04 |
| Lumbricillus lineatus | Annelida | 0.09 | 0.97 | 0.20 |
| Lumbrineris fragilis CMC01 | Annelida | 0.33 | 0.95 | 0.05 |
| Mallotus villosus | Chordata | 0.15 | 0.96 | 0.08 |
| Marenzelleria viridis | Annelida | 0.14 | 0.97 | 0.29 |
| Margarites helicinus | Mollusca | 0.14 | 0.92 | 0.11 |
| Melanothamnus harveyi |  | 0.41 | 0.97 | 0.25 |
| Membranipora membranacea | Bryozoa | 0.40 | 0.97 | 0.10 |
| Micromonas pusilla | Chlorophyta | 0.92 | 0.99 | 0.19 |
| Minutocellus polymorphus | Bacillariophyta | 0.29 | 0.95 | 0.05 |
| Mitrocomella polydiademata | Cnidaria | 0.19 | 0.95 | 0.04 |
| Modiolus modiolus | Mollusca | 0.34 | 0.95 | 0.05 |
| Monocorophium insidiosum | Arthropoda | 0.09 | 0.97 | 0.27 |
| Myoxocephalus aenaeus | Chordata | 0.20 | 0.96 | 0.11 |
| Myoxocephalus scorpius | Chordata | 0.16 | 0.96 | 0.18 |
| Myrionema strangulans | Phaeophyceae | 0.14 | 0.98 | 0.32 |
| Myriotrichia claviformis | Phaeophyceae | 0.28 | 0.97 | 0.21 |
| Mytilus edulis | Mollusca | 0.40 | 0.97 | 0.08 |
| Mytilus trossulus | Mollusca | 0.57 | 0.97 | 0.09 |
| Nanomia cara | Cnidaria | 0.35 | 0.95 | 0.07 |
| Nemalion multifidum |  | 0.14 | 0.97 | 0.29 |
| Nereis pelagica CMC02 | Annelida | 0.47 | 0.94 | 0.21 |
| Noctua pronuba | Arthropoda | 0.08 | 0.97 | 0.56 |
| Nucella lapillus | Mollusca | 0.09 | 0.97 | 0.22 |
| Obelia bidentata | Cnidaria | 0.18 | 0.95 | 0.05 |
| Obelia geniculata | Cnidaria | 0.52 | 0.95 | 0.04 |
| Octolasion cyaneum | Annelida | 0.15 | 0.96 | 0.08 |
| Oithona similis | Arthropoda | 0.92 | 0.99 | 0.93 |
| Onchidoris muricata | Mollusca | 0.29 | 0.95 | 0.05 |
| Ophiopholis aculeata | Echinodermata | 0.86 | 0.96 | 0.19 |
| Orchesella cincta | Arthropoda | 0.21 | 0.95 | 0.04 |
| Orthopyxis integra | Cnidaria | 0.16 | 0.94 | 0.22 |
| Pagurus acadianus | Arthropoda | 0.08 | 0.97 | 0.40 |
| Palmaria palmata |  | 0.27 | 0.91 | 0.44 |
| Pandalus montagui | Arthropoda | 0.23 | 0.95 | 0.04 |
| Parvicirrus dubius | Nemertea | 0.21 | 0.95 | 0.04 |
| Pemphigus bursarius | Arthropoda | 0.14 | 0.95 | 0.09 |
| Petalonia fascia | Phaeophyceae | 0.62 | 0.74 | 0.56 |
| Petalonia filiformis | Phaeophyceae | 0.26 | 0.94 | 0.46 |
| Petalonia zosterifolia | Phaeophyceae | 0.14 | 0.98 | 0.39 |
| Pholis gunnellus | Chordata | 0.23 | 0.87 | 0.67 |
| Phyllodoce groenlandica CMC01 | Annelida | 0.19 | 0.95 | 0.05 |
| Phyllodoce mucosa | Annelida | 0.19 | 0.95 | 0.05 |
| Phyllophora pseudoceranoides |  | 0.19 | 0.95 | 0.05 |
| Phymatolithon lenormandii |  | 0.18 | 0.96 | 0.12 |
| Phytophthora gonapodyides |  | 0.08 | 0.97 | 0.35 |
| Phytophthora undulata |  | 0.22 | 0.95 | 0.04 |
| Placida dendritica | Mollusca | 0.21 | 0.95 | 0.04 |
| Podon intermedius | Arthropoda | 0.32 | 0.96 | 0.03 |
| Polysiphonia fucoides |  | 0.16 | 0.96 | 0.08 |
| Porcellio scaber | Arthropoda | 0.19 | 0.95 | 0.04 |
| Porphyra corallicola |  | 0.18 | 0.96 | 0.15 |
| Porphyra umbilicalis |  | 0.14 | 0.98 | 0.58 |
| Pseudo-nitzschia delicatissima | Bacillariophyta | 0.37 | 0.95 | 0.04 |
| Pseudocalanus minutus | Arthropoda | 0.29 | 0.95 | 0.05 |
| Pseudocalanus newmani | Arthropoda | 0.92 | 0.99 | 0.38 |
| Pseudochattonella farcimen |  | 0.92 | 0.99 | 0.79 |
| Pseudopleuronectes americanus | Chordata | 0.17 | 0.97 | 0.14 |
| Pseudoscourfieldia marina | Chlorophyta | 0.92 | 0.99 | 0.60 |
| Ptilota serrata |  | 0.32 | 0.96 | 0.10 |
| Punctaria latifolia | Phaeophyceae | 0.31 | 0.97 | 0.11 |
| Pylaiella littoralis | Phaeophyceae | 0.29 | 0.96 | 0.05 |
| Pylaiella washingtoniensis | Phaeophyceae | 0.36 | 0.89 | 0.40 |
| Pyropia haitanensis |  | 0.15 | 0.96 | 0.23 |
| Ralfsia verrucosa | Phaeophyceae | 0.11 | 0.97 | 0.12 |
| Rathkea octopunctata | Cnidaria | 0.16 | 0.96 | 0.07 |
| Rhodomela lycopodioides |  | 0.14 | 0.98 | 0.34 |
| Rhodophysema elegans |  | 0.14 | 0.98 | 0.34 |
| Rubrointrusa membranacea |  | 0.15 | 0.96 | 0.06 |
| Saccharina latissima | Phaeophyceae | 0.29 | 0.96 | 0.14 |
| Saccorhiza dermatodea | Phaeophyceae | 0.21 | 0.95 | 0.04 |
| Salmo trutta | Chordata | 0.15 | 0.93 | 0.11 |
| Salvelinus fontinalis | Chordata | 0.21 | 0.95 | 0.04 |
| Sarsia tubulosa | Cnidaria | 0.15 | 0.96 | 0.21 |
| Scytosiphon canaliculatus | Phaeophyceae | 0.20 | 0.97 | 0.11 |
| Scytosiphon complanatus | Phaeophyceae | 0.16 | 0.97 | 0.17 |
| Scytosiphon lomentaria | Phaeophyceae | 0.48 | 0.78 | 0.55 |
| Semibalanus balanoides | Arthropoda | 0.15 | 0.98 | 0.23 |
| Sheathia americana |  | 0.19 | 0.95 | 0.04 |
| Skeletonema costatum | Bacillariophyta | 0.92 | 0.99 | 0.42 |
| Sminthurinus elegans | Arthropoda | 0.20 | 0.95 | 0.04 |
| Spongonema tomentosum | Phaeophyceae | 0.25 | 0.96 | 0.07 |
| Steatoda bipunctata | Arthropoda | 0.21 | 0.95 | 0.04 |
| Stictyosiphon tortilis | Phaeophyceae | 0.14 | 0.96 | 0.08 |
| Striaria attenuata | Phaeophyceae | 0.15 | 0.96 | 0.23 |
| Strongylocentrotus droebachiensis | Echinodermata | 0.92 | 0.96 | 0.28 |
| Strongylocentrotus pallidus | Echinodermata | 0.26 | 0.96 | 0.18 |
| Sus scrofa | Chordata | 0.22 | 0.96 | 0.03 |
| Synorthocladius semivirens | Arthropoda | 0.21 | 0.95 | 0.04 |
| Syrphus torvus | Arthropoda | 0.21 | 0.95 | 0.04 |
| Takayama acrotrocha |  | 0.87 | 0.98 | 0.18 |
| Tectura testudinalis | Mollusca | 0.10 | 0.97 | 0.13 |
| Telmatogeton japonicus | Arthropoda | 0.14 | 0.96 | 0.07 |
| Temora longicornis | Arthropoda | 0.24 | 0.97 | 0.15 |
| Tergipes tergipes | Mollusca | 0.10 | 0.97 | 0.16 |
| Thalassiosira nordenskioeldii | Bacillariophyta | 0.59 | 0.95 | 0.06 |
| Thalassiosira punctigera | Bacillariophyta | 0.92 | 0.98 | 0.11 |
| Thysanoessa raschii | Arthropoda | 0.54 | 0.95 | 0.04 |
| Tiaropsis multicirrata | Cnidaria | 0.31 | 0.95 | 0.05 |
| Tilopteris mertensii | Phaeophyceae | 0.18 | 0.95 | 0.04 |
| Tonicella rubra | Mollusca | 0.34 | 0.97 | 0.09 |
| Tortanus discaudatus | Arthropoda | 0.33 | 0.95 | 0.05 |
| Trachynema groenlandicum | Phaeophyceae | 0.15 | 0.97 | 0.21 |
| Ulvaria subbifurcata | Chordata | 0.18 | 0.97 | 0.14 |
| Uroleucon taraxaci | Arthropoda | 0.20 | 0.95 | 0.04 |
| Urophycis tenuis | Chordata | 0.14 | 0.96 | 0.08 |
| Valenzuela flavidus | Arthropoda | 0.15 | 0.96 | 0.08 |
| Zoarces americanus | Chordata | 0.24 | 0.95 | 0.04 |
