# Supplementary material for: Validating metabarcoding-based biodiversity assessments with multi-species occupancy models: A case study using coastal marine eDNA
Source: PLoS One. 2020 Mar 19;15(3):e0224119. doi: 10.1371/journal.pone.0224119 (PMC7082047; doi:10.1371/journal.pone.0224119)
Supplement: S1 File — Calculations presented for likelihood, Bayesian p-value, WAIC, CPO, Brier score and log score for model assessment and comparison. (DOCX) [file pone.0224119.s003.docx]

**S1 File. Model assessment calculations for multi-species, multi-scale occupancy models.** Calculations presented for likelihood, Bayesian p-value, WAIC, CPO, Brier score and log score for model assessment and comparison. All calculations were adapted from Broms et al. (2016) for a multi-scale model.

Ψ: probability of occupancy

ϴ: probability of capture

p: probability of detection

*k* represents the species to a total of K species.

*i* represents the site to a total of I sites.

*j* represents the biological replicate up to a total of J replicates. Biological replicates come from the same site.

*r* represents the technical replicate up to a total of R replicates. Technical replicates occur within each biological replicate.

*s* represents the number of MCMC samples up to S samples.

$I_{\left\{ \sum_{j=1}^{J_{i}} \sum_{r=1}^{R_{j}} y_{kijr}>0 \right\}}$ indicates sites whether a given species was detected at least once across all biological and technical replicates.

$I_{\left\{ \sum_{r=1}^{R_{j}} y_{kijr}>0 \right\}}$ indicates biological replicates where a given species was detected at least once across all technical replicates.

Integrated Likelihood

For 3 biological replicates (terms added to eqn. for more replicates)

$$\left[ y_{ki}|\psi_{ki}, \theta_{ki}, p_{ki} \right]=I_{\left\{ \sum_{j=1}^{J_{i}} \sum_{r=1}^{R_{j}} y_{kijr}>0 \right\}}\left( \prod_{j=1}^{J_{i}} I_{\left\{ \sum_{r=1}^{R_{j}} y_{kijr}>0 \right\}}\left( \psi_{ki}\theta_{kij}\prod_{r=1}^{R_{j}} {p_{kijr}}^{y_{kijr}}\left( 1-p_{kijr} \right)^{1-y_{kijr}} \right)+ \left( 1-I_{\left\{ \sum_{r=1}^{R_{j}} y_{kijr}>0 \right\}} \right)\left( \psi_{ki}\left( 1-\theta_{kij} \right)+ \psi_{ki}\theta_{kij}\prod_{r=1}^{R_{j}} \left( 1-p_{kijr} \right) \right) \right)+ \left( 1- I_{\left\{ \sum_{j=1}^{J_{i}} \sum_{r=1}^{R_{j}} y_{kijr}>0 \right\}} \right)\left( \left( 1- \psi_{ki} \right)+ \psi_{ki}\prod_{j=1}^{J_{i}} \left( 1-\theta_{kij} \right)+\psi_{ki}\prod_{j=1}^{J_{i}} \theta_{kij}\prod_{r=1}^{R_{j}} \left( 1-p_{kijr} \right)+\psi_{ki}\theta_{ki1}\theta_{ki2}\left( 1-\theta_{ki3} \right)\prod_{r=1}^{R_{j}} \left( 1-p_{ki1r} \right)\left( 1-p_{ki2r} \right)+\psi_{ki}\theta_{ki1}\left( 1-\theta_{ki2} \right)\theta_{ki3}\prod_{r=1}^{R_{j}} \left( 1-p_{ki1r} \right)\left( 1-p_{ki3r} \right)+\psi_{ki}\left( {1-\theta}_{ki1} \right)\theta_{ki2}\theta_{ki3}\prod_{r=1}^{R_{j}} \left( 1-p_{ki2r} \right)\left( 1-p_{ki3r} \right) +\psi_{ki}\left( {1-\theta}_{ki1} \right)\left( 1-\theta_{ki2} \right)\theta_{ki3}\prod_{r=1}^{R_{j}} \left( 1-p_{ki3r} \right) +\psi_{ki}\left( {1-\theta}_{ki1} \right)\theta_{ki2}\left( {1-\theta}_{ki3} \right)\prod_{r=1}^{R_{j}} \left( 1-p_{ki2r} \right) +\psi_{ki}\theta_{ki1}\left( 1-\theta_{ki2} \right)\left( {1-\theta}_{ki3} \right)\prod_{r=1}^{R_{j}} \left( 1-p_{ki1r} \right) \right)$$

Deviance (for Bayesian P-Value)

Ḋ and ẏ are associated with the predicted detections from the model.

$$D^{(s)}= -2 \sum_{k=1}^{K} \sum_{i=1}^{I} \log\left[ y_{ki}|\psi_{ki}^{\left( s \right)},\theta_{ki}^{(s)}, p_{ki}^{(s)} \right]$$

$$Ḋ^{(s)}= -2 \sum_{k=1}^{K} \sum_{i=1}^{I} \log\left[ ẏ_{ki}|\psi_{ki}^{\left( s \right)},\theta_{ki}^{(s)}, p_{ki}^{(s)} \right]$$

WAIC

$$WAIC=\left( -2*elppd \right)+(2*pD_{WAIC})$$

$$elppd \approx\sum_{k=1}^{K} \sum_{i=1}^{I} log\left( \frac{\sum_{s=1}^{S} \left[ y_{ki}|\psi_{ki}^{\left( s \right)}, ϴ_{ki}^{(s)}, p_{ki}^{(s)} \right]}{S} \right)$$

$$pD_{WAIC}\approx\sum_{k=1}^{K} \sum_{i=1}^{I} {Var}_{post}\left( log\left( \left[ y_{ki}| \psi_{ki}, ϴ_{ki},p_{ki} \right] \right) \right)$$

CPO

$$CPO= -\sum_{k=1}^{K} \sum_{i=1}^{I} log\left( {CPO}_{ij} \right)$$

$${CPO}_{ij}\approx\frac{S}{\sum_{s=1}^{S} \left[ y_{ki}| \psi_{ki}^{(s)},ϴ_{ki}^{(s)}p_{ki}^{(s)} \right]^{-1}}$$

Cross-Validation: Brier Score

$$L_{\mathrm{Brier}}= \frac{1}{k^{*}}\sum_{k^{*}} \frac{1}{S}\sum_{s=1}^{S} \left( \sum_{k=1}^{K} \sum_{i=1}^{I*} \sum_{j=1}^{J_{i}} \sum_{r=1}^{R_{j}} y_{kijr}^{*}\left( 1- \psi_{ki}^{(s)}\theta_{kij}^{(s)}p_{kijr}^{(s)} \right)^{2}+\left( 1-y_{kijr}^{*} \right)\left( \psi_{ki}^{(s)}\theta_{kij}^{(s)}p_{kijr}^{(s)} \right)^{2} \right)$$

Cross-Validation: Logarithmic Score

$$L_{\mathrm{Log}}=\frac{1}{k^{*}}\sum_{k^{*}} \frac{1}{S}\sum_{s=1}^{S} -2\left( \sum_{k=1}^{K} \sum_{i=1}^{I^{*}} \log\left[ y_{ki}^{*}|\psi_{ki}^{\left( s \right)},\theta_{ki}^{(s)}, p_{ki}^{(s)} \right] \right)$$

* no need to sum over sites if data is subsetted by sites for cross-validation
