# Supplementary material for: Validating metabarcoding-based biodiversity assessments with multi-species occupancy models: A case study using coastal marine eDNA
Source: PLoS One. 2020 Mar 19;15(3):e0224119. doi: 10.1371/journal.pone.0224119 (PMC7082047; doi:10.1371/journal.pone.0224119)
Supplement: S2 File — BUGS model structure presented for the most complex model ψ(water depth) ϴ(.) p(sequencing depth, amplicon), the best model ψ(water depth) ϴ(.) p(sequencing depth) with data augmentation and the ψ(.) ϴ(.) p(amplicon) model with a group-level effect (phylum). (DOCX) [file pone.0224119.s004.docx]

**S2 File. Model structure in BUGS language.** BUGS model structure presented for the most complex model ψ(water depth) ϴ(.) p(sequencing depth, amplicon), the best model ψ(water depth) ϴ(.) p(sequencing depth) with data augmentation and the ψ(.) ϴ(.) p(amplicon) model with a group-level effect (phylum).

**Model 2**

***ψ*(water depth) *ϴ*(.) *p*(sequencing depth, amplicon)**

model {

# Priors for species-specific effects in occupancy and detection

for(k in 1:nspec){

lpsi[k] ~ dnorm(mu.lpsi, tau.lpsi) # Hyperparameters describe community

betalpsi[k] ~ dnorm(mu.betalpsi, tau.betalpsi)

ltheta[k] ~ dnorm(mu.ltheta, tau.ltheta)

for (m in 1:ntechrep) {

betalp2[k,m] ~ dnorm(mu.betalp2, tau.betalp2)

}

lp[k] ~ dnorm(mu.lp, tau.lp)

betalp1[k] ~ dnorm(mu.betalp1, tau.betalp1)

}

# Hyperpriors

# For the model of occupancy

mu.lpsi ~ dnorm(0,0.01)

sd.lpsi ~ dunif(0,5) # bounds of uniform chosen by trial and error

tau.lpsi <- pow(sd.lpsi, -2)

mu.betalpsi ~ dnorm(0,0.01)

sd.betalpsi ~ dunif(0, 5)

tau.betalpsi <- pow(sd.betalpsi, -2)

# For the model of availability

mu.ltheta ~ dnorm(0,0.01)

sd.ltheta ~ dunif(0, 5)

tau.ltheta <- pow(sd.ltheta, -2)

# For the model of detection

mu.lp ~ dnorm(0,0.01)

sd.lp ~ dunif(0, 5)

tau.lp <- pow(sd.lp, -2)

mu.betalp1 ~ dnorm(0,0.01)

sd.betalp1 ~ dunif(0, 5)

tau.betalp1 <- pow(sd.betalp1, -2)

mu.betalp2 ~ dnorm(0,0.01)

sd.betalp2 ~ dunif(0, 5)

tau.betalp2 <- pow(sd.betalp1, -2)

# Ecological model for true occurrence (process model)

for(k in 1:nspec){

for (i in 1:nsite) {

logit(psi[i,k]) <- lpsi[k] + betalpsi[k] * depth[i]

z[i,k] ~ dbern(psi[i,k])

# asim, bsim and csim generate a simulated community using parameters from each iteration

# used to calculate Bayesian p-value

asim[i,k] ~ dbern(psi[i,k]) # asim, bsim and csim generate a simulated community

}

}

# Observation model for replicated detection/non-detection observations

for(k in 1:nspec){

for (i in 1:nsite){

for(j in 1:nbiorep){

logit(theta[i,j,k]) <- ltheta[k]

mu.theta[i,j,k] <- z[i,k] * theta[i,j,k]

w[i,j,k] ~ dbern(mu.theta[i,j,k])

bsim[i,j,k] ~ dbern(asim[i,k]*theta[i,j,k])

}

}

}

# Observation model for replicated amplicons for each sample

for(k in 1:nspec){

for (i in 1:nsite){

for(j in 1:nbiorep){

for (r in 1:ntechrep){

logit(p[i,j,r,k]) <- lp[k] + betalp2[k,amplicon[i,j,r]] + betalp1[k] * seq[i,j,r]

mu.p[i,j,r,k] <- w[i,j,k] * p[i,j,r,k]

y[i,j,r,k] ~ dbern(mu.p[i,j,r,k])

csim[i,j,r,k] ~ dbern(bsim[i,j,k] * p[i,j,r,k])

}

}

}

}

}

**Model 7 with data augmentation**

***ψ*(water depth) *ϴ*(.) *p*(sequencing depth)**

model {

# Priors

omega ~ dunif(0,1)

# Priors for species-specific effects in occupancy and detection

for(k in 1:M){

lpsi[k] ~ dnorm(mu.lpsi, tau.lpsi) # Hyperparameters describe community

betalpsi[k] ~ dnorm(mu.betalpsi, tau.betalpsi)

ltheta[k] ~ dnorm(mu.ltheta, tau.ltheta)

lp[k] ~ dnorm(mu.lp, tau.lp)

betalp1[k] ~ dnorm(mu.betalp1, tau.betalp1)

}

# Hyperpriors

# For the model of occupancy

mu.lpsi ~ dnorm(0,0.01)

sd.lpsi ~ dunif(0,5) # bounds of uniform chosen by trial and error

tau.lpsi <- pow(sd.lpsi, -2)

mu.betalpsi ~ dnorm(0,0.01)

sd.betalpsi ~ dunif(0, 5)

tau.betalpsi <- pow(sd.betalpsi, -2)

# For the model of availability

mu.ltheta ~ dnorm(0,0.01)

sd.ltheta ~ dunif(0, 5)

tau.ltheta <- pow(sd.ltheta, -2)

# For the model of detection

mu.lp ~ dnorm(0,0.01)

sd.lp ~ dunif(0, 5)

tau.lp <- pow(sd.lp, -2)

mu.betalp1 ~ dnorm(0,0.01)

sd.betalp1 ~ dunif(0, 5)

tau.betalp1 <- pow(sd.betalp1, -2)

# Superpopulation process: Ntotal species sampled out of M available

for(k in 1:M){

a[k] ~ dbern(omega)

}

# Ecological model for true occurrence (process model)

for(k in 1:M){

for (i in 1:nsite) {

logit(psi[i,k]) <- lpsi[k] + betalpsi[k] * depth[i]

mu.psi[i,k] <- a[k] * psi[i,k]

z[i,k] ~ dbern(mu.psi[i,k])

}

}

# Observation model for replicated detection/non-detection observations

for(k in 1:M){

for (i in 1:nsite){

for(j in 1:nbiorep){

logit(theta[i,j,k]) <- ltheta[k]

mu.theta[i,j,k] <- z[i,k] * theta[i,j,k]

w[i,j,k] ~ dbern(mu.theta[i,j,k])

}

}

}

# Observation model for replicated amplicons for each sample

for(k in 1:M){

for (i in 1:nsite){

for(j in 1:nbiorep){

for (r in 1:ntechrep){

logit(p[i,j,r,k]) <- lp[k] + betalp1[k] * seq[i,j,r]

mu.p[i,j,r,k] <- w[i,j,k] * p[i,j,r,k]

y[i,j,r,k] ~ dbern(mu.p[i,j,r,k])

}

}

}

}

# Derived quantities

n0 <- sum(a[(nspec+1):(nspec+nz)]) # Number of unseen species

Ntotal <- sum(a[]) # Total metacommunity size

}

**Model 4 with Phylum as group-effect**

***ψ*() *ϴ*(.) *p*(amplicon)**

model {

# Priors for species-specific effects in occupancy and detection

# Hyperparameters vary by group (phylum)

for(k in 1:nspec){

lpsi[k] ~ dnorm(mu.lpsi[phylum[k]], tau.lpsi[phylum[k]])

ltheta[k] ~ dnorm(mu.ltheta[phylum[k]], tau.ltheta[phylum[k]])

for (m in 1:ntechrep) {

betalp2[k,m] ~ dnorm(mu.betalp2[phylum[k]], tau.betalp2[phylum[k]])

}

lp[k] ~ dnorm(mu.lp[phylum[k]], tau.lp[phylum[k]])

}

# Hyperpriors

for (g in 1:nphylum) {

# For the model of occupancy

mu.lpsi[g] ~ dnorm(0,0.01)

sd.lpsi[g] ~ dunif(0,5) # bounds of uniform chosen by trial and error

tau.lpsi[g] <- pow(sd.lpsi[g], -2)

# For the model of availability

mu.ltheta[g] ~ dnorm(0,0.01)

sd.ltheta[g] ~ dunif(0, 5)

tau.ltheta[g] <- pow(sd.ltheta[g], -2)

# For the model of detection

mu.lp[g] ~ dnorm(0,0.01)

sd.lp[g] ~ dunif(0, 5)

tau.lp[g] <- pow(sd.lp[g], -2)

mu.betalp2[g] ~ dnorm(0,0.01)

sd.betalp2[g] ~ dunif(0, 5)

tau.betalp2[g] <- pow(sd.betalp2[g], -2)

}

# Ecological model for true occurrence (process model)

for(k in 1:nspec){

for (i in 1:nsite) {

logit(psi[i,k]) <- lpsi[k]

z[i,k] ~ dbern(psi[i,k])

}

}

# Observation model for replicated detection/non-detection observations

for(k in 1:nspec){

for (i in 1:nsite){

for(j in 1:nbiorep){

logit(theta[i,j,k]) <- ltheta[k]

mu.theta[i,j,k] <- z[i,k] * theta[i,j,k]

w[i,j,k] ~ dbern(mu.theta[i,j,k])

}

}

}

# Observation model for replicated amplicons for each sample

for(k in 1:nspec){

for (i in 1:nsite){

for(j in 1:nbiorep){

for (r in 1:ntechrep){

logit(p[i,j,r,k]) <- lp[k] + betalp2[k,amplicon[i,j,r]]

mu.p[i,j,r,k] <- w[i,j,k] * p[i,j,r,k]

y[i,j,r,k] ~ dbern(mu.p[i,j,r,k])

}

}

}

}

}
