# Supplementary material for: Validating metabarcoding-based biodiversity assessments with multi-species occupancy models: A case study using coastal marine eDNA
Source: PLoS One. 2020 Mar 19;15(3):e0224119. doi: 10.1371/journal.pone.0224119 (PMC7082047; doi:10.1371/journal.pone.0224119)
Supplement: S3 File — Plots of residual deviance for each species, site and covariate from a multi-species, multi-scale occupancy model based on eDNA metabarcoding data collected from Conception Bay, Newfoundland. (DOCX) [file pone.0224119.s005.docx]

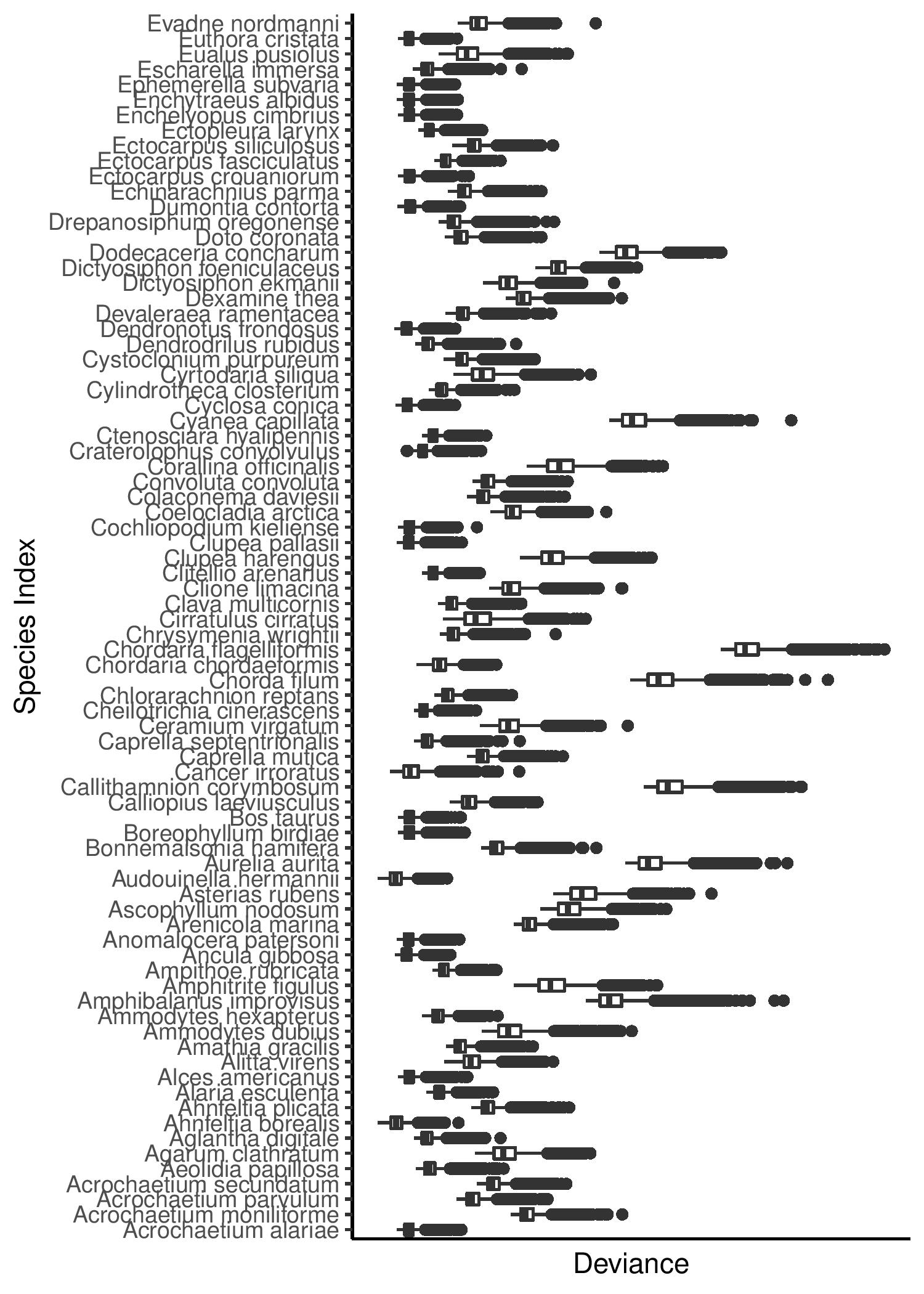
**S3 File. Diagnostic plots to asses fit of ψ(water depth) ϴ(.) p(sequencing depth) model.** Plots of residual deviance for each species, site and covariate from a multi-species, multi-scale occupancy model based on eDNA metabarcoding data collected from Conception Bay, Newfoundland.


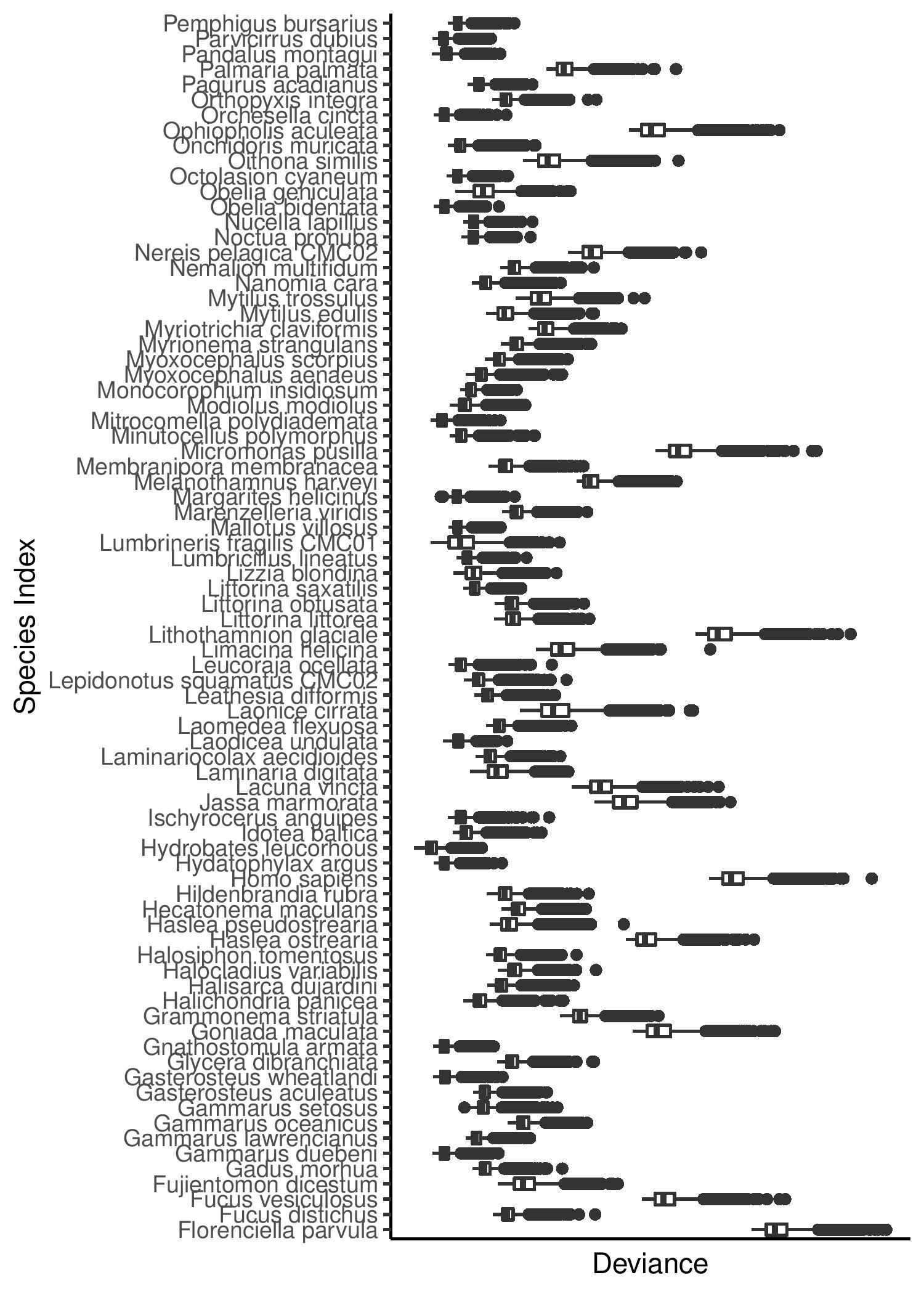


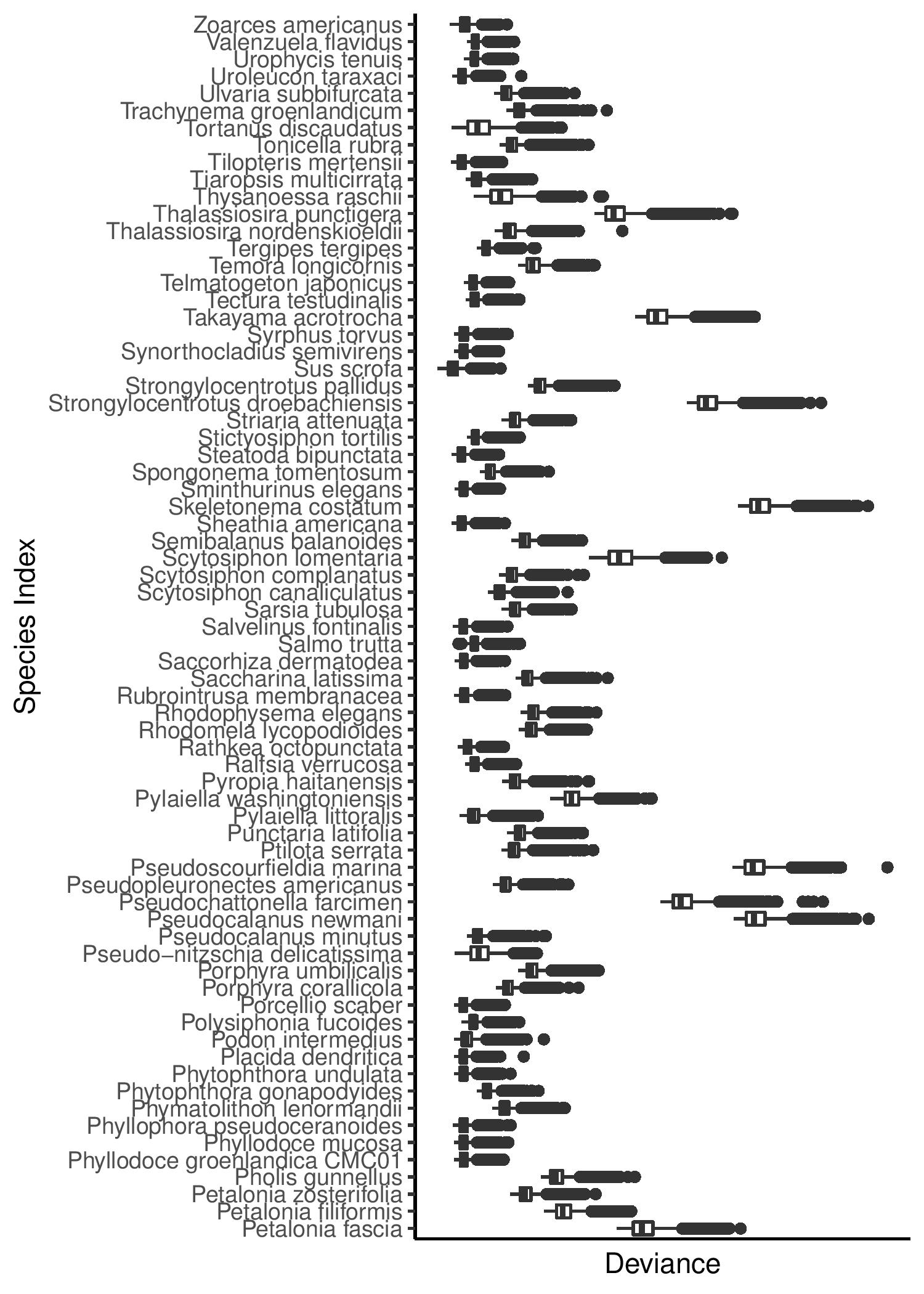


Figure 1 - Plot of residual deviance for each species from a multi-species, multi-scale occupancy model based on eDNA metabarcoding data collected from Conception Bay, Newfoundland. The band in the middle of the box represents the median and the upper and lower edges of the box represent the upper and lower quartiles. The whiskers represent 1.5 times the inter-quartile range.


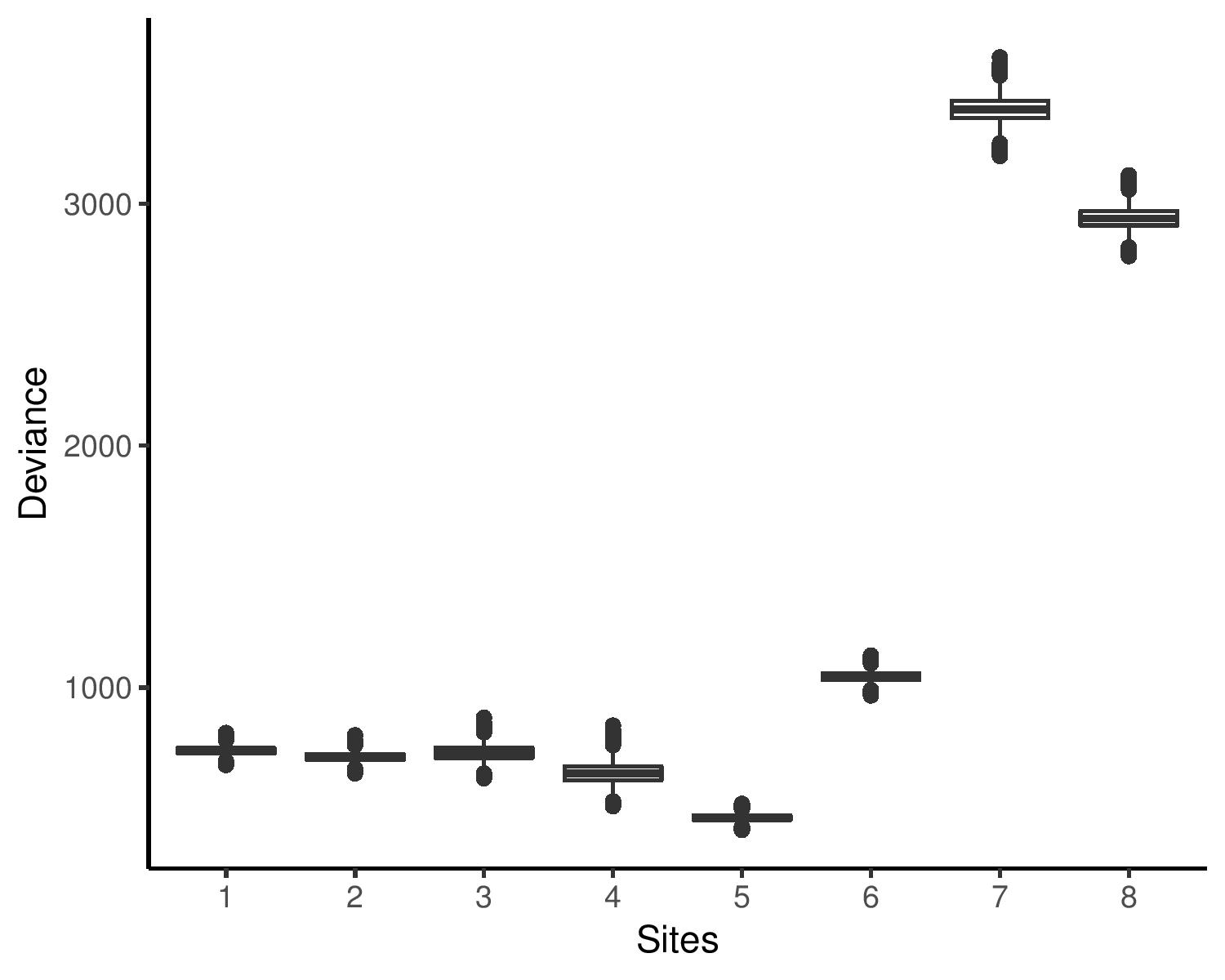


Figure 2 - Boxplot of residual deviance for each site from a multi-species, multi-scale occupancy model based on eDNA metabarcoding data collected from Conception Bay, Newfoundland. The band in the middle of the box represents the median and the upper and lower edges of the box represent the upper and lower quartiles. The whiskers represent 1.5 times the inter-quartile range.


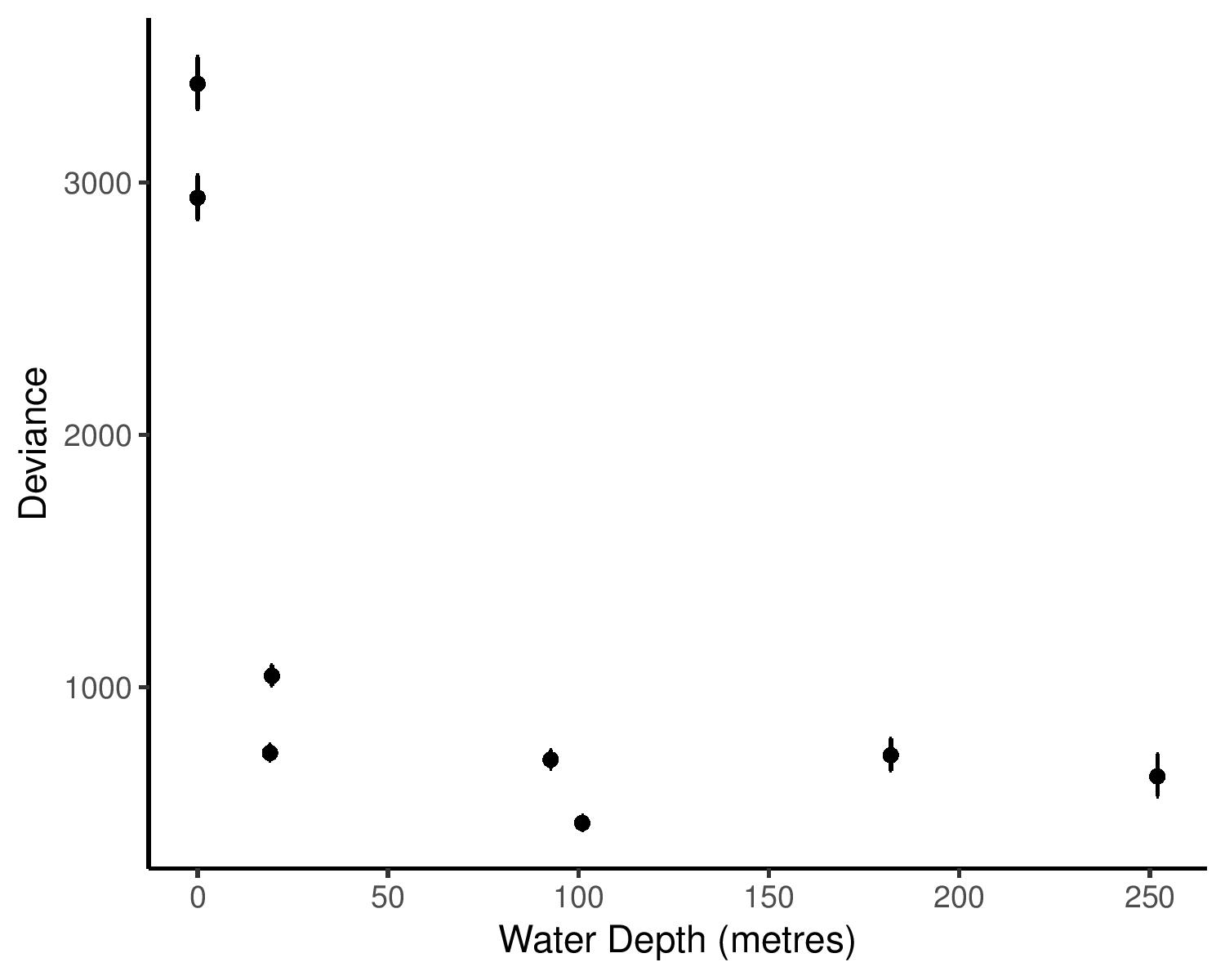


Figure 3 - Residual deviances on occupancy and water depth. Error bars represent 95% confidence intervals.
